# Immunoglobulin genes expressed in lymphoblastoid cell lines discern and predict lithium response in bipolar disorder patients

**DOI:** 10.1038/s41380-023-02183-z

**Published:** 2023-07-24

**Authors:** Liron Mizrahi, Ashwani Choudhary, Polina Ofer, Gabriela Goldberg, Elena Milanesi, John R. Kelsoe, David Gurwitz, Martin Alda, Fred H. Gage, Shani Stern

**Affiliations:** 1 Sagol Department of Neurobiology, Faculty of Natural Sciences, University of Haifa, Haifa, 3498838 Israel; 2 Laboratory of Genetics, The Salk Institute for Biological Studies, La Jolla, CA 92037 USA; 3 Victor Babes National Institute of Pathology, Bucharest, 050096 Romania; 4 Department of Psychiatry, University of California, San Diego, La Jolla, CA 92093 USA; 5 Department of Human Molecular Genetics and Biochemistry, Faculty of Medicine, Tel Aviv University, Tel Aviv, 69978 Israel; 6 Department of Psychiatry, Dalhousie University, Halifax, NS B3H 2E2 Canada

**Keywords:** Neuroscience, Predictive markers, Bipolar disorder

## Abstract

Bipolar disorder (BD) is a neuropsychiatric mood disorder manifested by recurrent episodes of mania and depression. More than half of BD patients are non-responsive to lithium, the first-line treatment drug, complicating BD clinical management. Given its unknown etiology, it is pertinent to understand the genetic signatures that lead to variability in lithium response. We discovered a set of differentially expressed genes (DEGs) from the lymphoblastoid cell lines (LCLs) of 10 controls and 19 BD patients belonging mainly to the immunoglobulin gene family that can be used as potential biomarkers to diagnose and treat BD. Importantly, we trained machine learning algorithms on our datasets that predicted the lithium response of BD subtypes with minimal errors, even when used on a different cohort of 24 BD patients acquired by a different laboratory. This proves the scalability of our methodology for predicting lithium response in BD and for a prompt and suitable decision on therapeutic interventions.

## Introduction

Bipolar disorder (BD) is a chronic, heritable neuropsychiatric disorder with alternating episodes of mania and depression in patients [1–3]. With a prevalence of 1–2 % in the general population, BD is also a notable cause of disability and premature deaths due to suicide or co-morbidities [2, 4]. Clinical management of BD is dependent on lithium and classes of antipsychotic and anticonvulsant drugs [5]. Due to its effectiveness, lithium is used as a first-line treatment for BD patients [3, 6, 7]. However, more than half of BD patients respond to lithium treatment inadequately; therefore, BD patients have been classified and characterized as lithium responders (LRs) and non-responders (NRs) [7, 8]. The presence of subpopulations among BD patients further hints at the confounding genetic complexity and heterogeneity among the patients. Some studies have also pointed toward the neurodevelopmental origin of BD, although the clinical symptoms are only visible in adulthood [8]. Genome-wide association studies (GWAS) in BD patients have identified genomic loci associated with BD but, in the absence of functional validation, the causative genes are obscure [9–13].

Animal models for BD are imperfect mainly due to the unknown genetic component and inability to mimic the extreme mood shifts observed in patients [14]. Disease modeling via induced pluripotent stem cell (iPSC) technology has opened a new possibility for studying psychiatric diseases by allowing researchers to investigate patient-specific neural cells in vitro in 2D and 3D culture systems [12]. Recently, researchers have employed iPSC technology to study BD patient-specific neurons to understand the cellular pathophysiology and mechanisms underlying BD [15–17]. In 2018, we found that patch clamp recordings of neurons derived from BD patients could be used to predict lithium response with a very low error rate [16]. While these investigations were expensive and well-trained personnel were needed to perform neuronal differentiation and electrophysiological experiments, they showed a proof of concept that the prediction of lithium response was indeed feasible with a low error rate. In addition to iPSCs, LCLs have been used to study drug response in BD. LCLs are immortal lymphoblastoid cell lines derived by the transformation of B lymphocytes by Epstein-Barr virus (EBV) [12, 18]. LCLs can expand indefinitely and are low-cost, patient-specific polyclonal cells as compared to patient-specific iPSCs, which are generally monoclonal and require higher cost and expertise to study [18].

Poor understanding of the etiology and genetics of BD also makes its diagnosis difficult, in turn affecting the therapy received by the patients. Hence, appropriate biomarkers are necessary to diagnose BD correctly as well as predict the patients’ response to lithium [19, 20]. Previous studies have found serum and plasma levels of BDNF; the altered response of lymphocytes to glucose deprivation; and oxidative stress markers to be potential biomarkers of BD [21–23]. Gene expression analysis using DNA microarrays from whole blood RNA of BD patients has found some candidate genes that are especially related to myelination or growth factor signaling [24]. While iPSCs offer an excellent system to study the cellular pathology and molecular mechanisms of the disease, due to their inexpensive nature LCLs are a good source to look for genetic biomarkers, especially in a large sample size including BD patient subtypes [24]. There are hypotheses linking an immune response with psychiatric disorders [25, 26], further suggesting that LCLs will have a predictive value. Earlier studies using LCLs from BD patients have reported altered calcium signaling and lower ER stress response in patients as compared to controls [12, 27, 28]. Subsequently, several studies have reinforced the idea of conducting gene expression analysis in the LCLs of BD patients to identify potential transcriptomic biomarkers [29–32].

Recently, machine-learning (ML) algorithms have found widespread applications in the field of healthcare [16, 33, 34], with the potential to provide effective support in clinical management through disease prediction, detection, and diagnosis [35]. Supervised classification algorithms are a type of ML approach that can be utilized as significant tools in the diagnosis and treatment decisions in complex disorders such as BD [36, 37]. We hypothesized that by analysing transcriptomic datasets with supervised classification algorithms, patterns of gene expression and biomarkers can be identified that are associated with BD and its subtypes. This information can be further used to train models and thus can be utilized for the prediction of treatment response in BD.

**Fig. 1:**
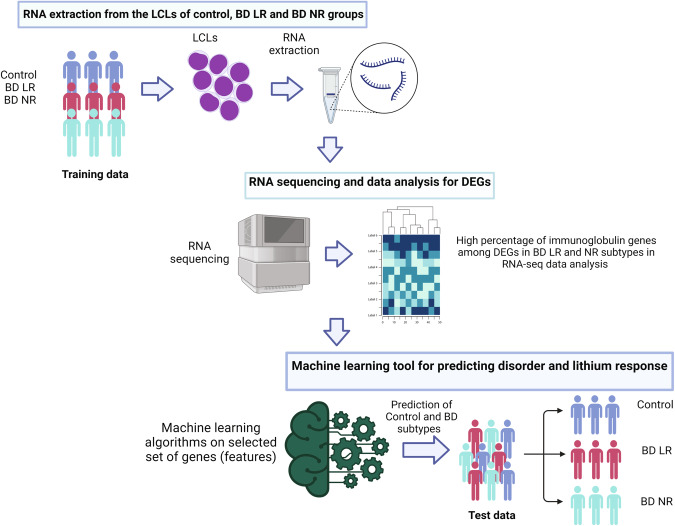
Graphical representation of the workflow and the experimental design. RNA sequencing was performed from the LCLs of Control and BD subtypes from the train data to find genetic signatures using machine learning algorithms to discern between Control, BD, and its subtypes and used it for classification of test data (Lithium Responsive (LR) and Lithium Non-responsive (NR)). The graphical image was created with Biorender.com.

In this study, we have performed RNA-seq of LCLs from control and BD patients, including LRs and NRs, and thereby report the analysis of DEGs to find appropriate genetic signatures that can be used as potential biomarkers in BD. We have also incorporated the RNA-seq data previously published by Milanesi et al. [38]. for BD LR and BD NR patient subtypes and analysed them jointly to add more statistical power to our results. Importantly, we trained supervised classification algorithms on the RNA-seq data, which predicted the lithium response from the RNA-seq data acquired by a different laboratory with a very low error rate (Fig. 1). Such validation is necessary since we are seeking global biomarkers that will work in algorithms that are trained in one laboratory and predictions need to be made based on data that are acquired elsewhere. Our study also found convergence in altered genes with earlier studies, providing insights into the important biological pathways affected in BD.

## Methods

### Ethics approval

All participants signed informed consent. The study was approved by the Research Ethics Board of the Nova Scotia Health Authority, Canada. The participants were diagnosed with BD and classified into subtypes according to the lithium response by the psychiatrists as described previously [16].

### Cell culture and RNA extraction from LCLs

LCLs were generated from the PBMCs of all the patients and controls (see details about the cohorts below) as described previously [39]. Briefly, PBMCs were isolated from the blood using BD Vacutainer® CPT™ Cell Preparation Tubes with Sodium Citrate (Cat no: 362761) following the manufacturer’s protocol. Approximately, 2 million cells were infected with the EBV produced from the B95-8 cell line as mentioned in detail previously [39, 40]. The transformed cells formed aggregates within a week of EBV infection and formed larger aggregates with time. These cells were further passaged 3-4 times to ascertain the establishment of lymphoblastoid lines (Supplementary Fig 1). The LCLs were cultured in T25 and T75 tissue culture flasks in complete RPMI medium containing RPMI 1640 (Biological Industries, Cat no: 01-100-1 A), 1X Anti-Anti (Thermofisher Scientific, Cat no: 15240062) 1% Glutamax (Thermofisher Scientific, Cat no: 35050061), 1% Sodium pyruvate (Thermofisher Scientific, 11360070) and 15% heat-inactivated FBS (Sigma, Cat no: F9665) as described previously [40, 41] with media changes on alternative days. To ascertain the confluency, the LCLs were counted using Trypan blue exclusion assay with Bio-Rad TC20™ automated cell counter following the manufacturer’s protocol and were passaged regularly when they reached a density of 200,000 cells/ml. All the cell lines used were tested using a Hy-Mycoplasma PCR kit (Hylabs, Cat No. KI 5034I) and found to be free of mycoplasma contamination.

For RNA extraction, approximately 10 million cells were collected in 1 ml of TRIzol™ reagent (Thermofisher Scientific, Cat no: 15596026) and placed on ice for 5 min before storing at −80 ^o^C. Total RNA was isolated using the Zymo Quick RNA kit (Zymo Research, Cat no: R1054) according to the manufacturer’s protocol. The RNA was further purified using the RNA clean and concentrator kit (Zymo Research, Cat no: R1013) following the manufacturer’s instructions. The RNA quality and integrity were checked using an ND-1000 Nanodrop spectrophotometer (Thermofisher Scientific) and Tape station 2200 (Agilent). All the RNA samples sent for sequencing had RNA integrity number values > 9.

### First cohort

We performed RNA sequencing from the LCLs of 9 LR (Lithium responsive) BD patients (15 sets of RNA samples), 10 BD NR (Lithium Non-responsive) patients (17 sets of RNA samples), and 10 control individuals (14 sets of RNA samples) grown and sequenced in 3 batches. Some of the samples had replicates and hence a total of 46 sets of RNA were sequenced and analyzed. (Details about the samples in the Github file link https://github.com/Precision-Disease-Modeling-Lab/Lithum-Respose-Predictor/blob/main/ML/LRvsNR/samples.csv). 24 samples (5 LR in duplicates, 4 NR in duplicates, 3 control in duplicates) in Batch 1; 10 samples (1 LR in duplicate, 2 NR in duplicate, 2 control in duplicate) in Batch 2; and 12 samples in Batch 3 (3 LR-no replicate, 1 NR in duplicate plus 4 NR, 4 control-no replicate). We have used batch correction to account for the differences that arise due to the batch effect as described below.

### Second cohort

The data for the second cohort were obtained from the published work of Milanesi et al. [38]. and consisted of 12 BD LR (12 sets of RNA samples–no replicates) and 12 BD NR (12 sets of RNA samples-no replicates) patients for which the RNA-sequencing was performed from LCLs. The LCLs used by Milanesi et al. [38]. were also generated using a similar method i.e. by infecting the PBMCs with Epstein Barr Virus produced from the B95-8 cell line. Similarly, a batch correction algorithm was performed as described below.

### RNA sequencing, analysis, and batch correction

Libraries for the LCL RNAs from BD and control subjects were prepared using a TruSeq RNA Library Prep Kit v2 (Illumina) following the manufacturer’s instructions. Quality control of the raw FASTQ files was performed using FastQC [42] (v0.11.5). Sequencing reads were aligned to the human genome (GRCh38.104) and quantified using STAR [43] (v2.7.9a). The R-based Bioconductor package DESeq2 [44] (v1.34.0) was used to perform differential gene expression analysis. The experimental design was modeled to consider batch and condition (~batch + condition). Batch-effect was accounted for by either including batch as a covariate in the linear model (allowing for correct estimation of degrees of freedom) or by using the remove batch effect function from the limma [45] package in R for Principal Component Analysis (PCA) and correlation analysis. This function uses linear modeling to estimate and reduce batch effects from gene expression data.

To account for the false positives when identifying DEGs, we performed the false discovery Rate (FDR) analysis. In DESeq2, the FDR is calculated using the Benjamini-Hochberg (BH) procedure, which adjusts the p-values of the hypothesis tested for multiple comparisons [46, 47]. In this study, for each pairwise comparison, genes that reached BH false discovery rate (FDR) (*p*-Adjusted) < 0.05 and log_2_fold-change ≥|1| were considered significant DEGs.

### Machine learning predictor analysis

The ML predictor analysis was performed using Python-based packages. For the predictor analysis, we used PyCombat [48] (Python-based package) to correct for the batch effect in the RNA sequencing data (that was different from the R-based package for batch correction used during DEG analysis as mentioned above). PyCombat uses the empirical Bayes method to adjust for the batch effects [48]. The empirical Bayes method is effective even with smaller sample sizes (>10) [49].

We evaluated the following five supervised classifiers: Logistic regression (Lr), Random Forest (RF), K-Nearest Neighbors (K-NN), Support Vector Machine (SVM), and Neural Network (NN). Among the classifiers selected for predictor analysis, RF is a well-known ensemble learning algorithm that combines the predictions of multiple decision trees trained on randomly selected subsets of the training data and features [35, 36, 50]. It can achieve high accuracy for a wide range of classification tasks, but may not be the best choice for applications requiring interpretability, scalability, or fast training times [35, 51–53]. For such applications, Lr, SVM, and NN may be better suited depending on the specific requirements [52, 53]. K-NN is a simple and non-parametric supervised learning algorithm that makes predictions based on the proximity and similarity to other data points in the training set [54, 55]. However, it can be computationally expensive and sensitive to the number of neighbor data points (or K parameter) [55]. NN can handle complex problems and a wide range of data types including high dimensional datasets but can also be computationally expensive, difficult to interpret, and prone to overfitting [56, 57]. Lr, another important classifier, is particularly useful for binary classification where interpretability and simplicity are important [35, 57]. However, it may not perform as well as other complex models such as NN or RF for tasks that involve complex nonlinear relationships. SVM can be used for high-dimensional data and nonlinear relationships but can be computationally expensive and sensitive to hyper-parameters required for learning [35, 55].

To avoid overfitting, a feature selection was performed to reduce the number of genes used for training the classifier. To this end, a Mann-Whitney U test was performed between the two groups using the datasets (batches 1–4). (The dataset had 39,000 genes. We dropped the genes with less than 10 counts after which a total of 22000 genes were kept for analysis). Twenty genes (out of the total 22,000 genes) with the smallest p-value were selected (Supplementary Fig 5,6). An exhaustive search with all gene combinations was then performed, and the subset of genes with the best chance to be used as a feature list in Lr was identified. From all gene combinations, only those with an accuracy score above 0.93 were selected. The 7 potential and promising genes (for LR vs. NR) and 5 promising genes (for BD vs. CTRL), which constantly appeared in the gene combination with high accuracy scores, were chosen as features (see Supp. Fig. 5b & 6b the prediction accuracy as a function of the number of features selected out of the chosen 20 genes). The selected features were used to train the classifiers using another Python-based package, sci-kit-learn [58]. We then performed cross-validation by splitting the data randomly into a 50% train and a 50% test, and the classification was repeated 50 times with these random selections (BD vs. CTRL and LR vs. NR). For BD vs. control, a total of 29 subjects (19 BD + 10 controls) from the original dataset were used. For LR vs. NR, a total of 43 subjects (19 BD subjects from the first cohort and 24 BD subjects from the second cohort were used). In addition to the cross-validation method which uses a shuffle train/test split from the entire dataset, we also performed a predictor evaluation for LR vs. NR by training the model with our in-house (original) datasets (the first 3 batches) and performing the classification on the second cohort (the different dataset from another laboratory) labeled as the batch 4 dataset [38].

To further evaluate the validity of this approach, we tested the performance of all 5 supervised classification algorithms that used the provided input features (5&7 for disorder and lithium response prediction respectively). For a binary classification problem, the resulting model decisions could fall into 4 categories: true positives (t_p_) when the model correctly predicts the positive class, erroneous positive predictions (false positives, f_p_), and, analogously, true negatives (t_n_) and false negatives (f_n_). The accuracy, the Receiver Operator Characteristic (ROC), and the confusion matrix were evaluated, and a cross-validation method was used to ensure the robustness of the classification. The results were averaged, or aggregated in the case of the confusion matrix, to create a span of results for each iteration. We used the Area under the Curve (AUC) of the ROC and the confusion matrix to assess the performance of the classifiers using the following metrics: Accuracy, Precision, and the AUC of the ROC:

1 $${{{{{{{\mathrm{accuracy}}}}}}}}\left( {y,\hat y} \right) = \frac{1}{{{{{{{{{\mathrm{n}}}}}}}}_{{{{{{{{\mathrm{samples}}}}}}}}}}}\mathop {\sum}\limits_{i = 0}^{{{{{{{{\mathrm{n}}}}}}}}_{{{{{{{{\mathrm{samples}}}}}}}}} - 1} {1(\hat yi = yi)}$$

2 $${{{{{{{\mathrm{precision}}}}}}}} = \frac{{tp}}{{tp{{{{{{{\mathrm{ + }}}}}}}}fp}}$$

3 $${{{{{{{\mathrm{recall}}}}}}}} = \frac{{tp}}{{tp + fn}}$$

## Results

### Differentially expressed genes between LR and NR LCLs are enriched in Immunoglobulin genes

**Fig. 2:**
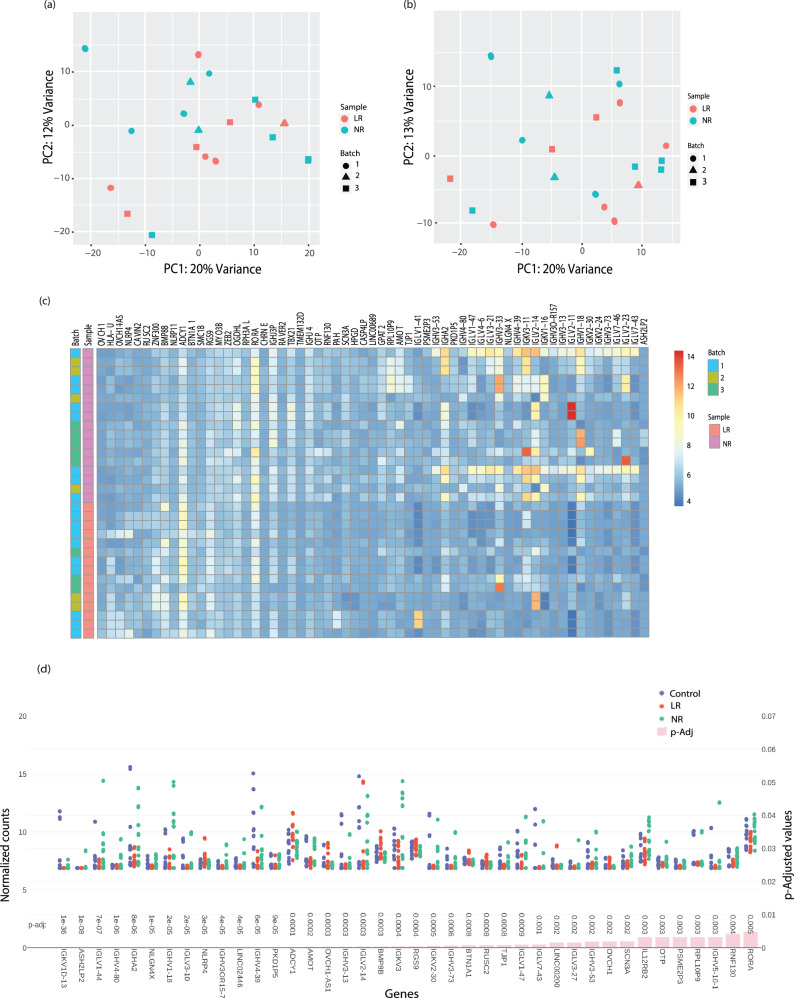
Differentially expressed genes between LR and NR LCLs are enriched in Immunoglobulin genes. **a** A PCA analysis of LR vs. NR LCLs’ RNA in the original (in-house) dataset before batch correction. Please note different colors are assigned for LR vs. NR & different shapes are assigned for different batches 1–3. LR RNA samples: 15; NR RNA samples: 17. 24 samples (5 LR in duplicates, 4 NR in duplicates, 3 control in duplicates) in Batch 1; 10 samples (1 LR in duplicate, 2 NR in duplicate, 2 control in duplicate) in Batch 2; and 12 samples in Batch 3 (3 LR-no replicate, 1 NR in duplicate plus 4 NR, 4 control-no replicate). In this figure, only LR and NR are plotted. **b** Same as (**a**) but after batch correction. **c** A heat map of the 61 DEGs (out of a total of 117 detected genes with FDR < 0.05) that reached a significance of FDR < 0.01 and fold-change >2 between BD LR and NR subtypes in their respective samples in the original dataset. (LR RNA samples: 15; NR RNA samples: 17. RNA samples were sequenced in replicates for some of the subjects as mentioned above). **d** The plot counts of the top ~40 significant DEGs between BD LR and NR samples (as mentioned above) compared to controls arranged according to the ascending order of FDR (*p*-Adjusted) values < 0.05 with a fold-change >2 in the original dataset (batches 1–3). The upper part of the graph shows the gene expression counts while the lower part of the graph presents the FDR values. The remaining DEGs have been plotted in Supplementary Fig 2a & b. (LR RNA samples: 15; NR RNA samples: 17. The expression counts have been normalized in DEseq2 in the Log2 scale).

To seek biomarkers for lithium response (Fig. 1.), we performed RNA sequencing of the LCLs of 19 BD patients. Nine of them were LRs and ten belonged to the NR subtype of BD. Since the LCLs were cultured and sequenced in three different batches (first cohort, batches1-3), we examined them for batch effects. Batch effects refer to the unwanted variations in the data that can arise due to technical differences, such as differences in sample preparation, sequencing, or batch processing. We thus performed a sensitivity analysis (PCA) to evaluate the impact of batch effects on the downstream analysis. We found the variability arising in PC1 associated with the batches as visualized in the PCA plot (Fig. 2a). For example, the second batch (triangles) can be seen concentrated near to first quadrant (upper right) of the PCA plot and the third batch (squares) are in the lower part (quadrants 3&4) in the PCA plot (Fig. 2a) regardless of the sample identity (LR or NR). We, therefore, performed a batch correction (see Methods) to account for this technical variability. Figure 2b shows the PCA after the batch correction and reduction of the unwanted variation in the RNA-seq dataset. We then performed differential gene expression analysis (see Methods) between the subtypes (LR vs. NR) of BD subjects. The sixty-one DEGs (out of approx. 117 genes with FDR < 0.05) with a fold change above 2 and an FDR < 0.01 between the LR and NR subtypes across different samples in different batches (batches 1–3) are presented in Fig. 2c as a heatmap. The extensive list of DEGs (approximately 117 genes with FDR < 0.05) with the plot counts has been plotted in Fig. 2d and Supplementary 2a & b in ascending order of FDR (*p*-Adj) values. Among the top 10 DEGs with the lowest p-Adj values, 70% belonged to the immunoglobulin heavy and light chain genes (Fig. 2d). A volcano plot is also shown in Supplementary Fig 2c. for the highly significant DEGs (*p*-Adj < 0.05) with a fold change of above 2 between LR vs. NR BD subtypes.

### Differentially expressed genes between LR and NR LCLs are highly enriched in Immunoglobulin genes when adding a dataset from another study

**Fig. 3:**
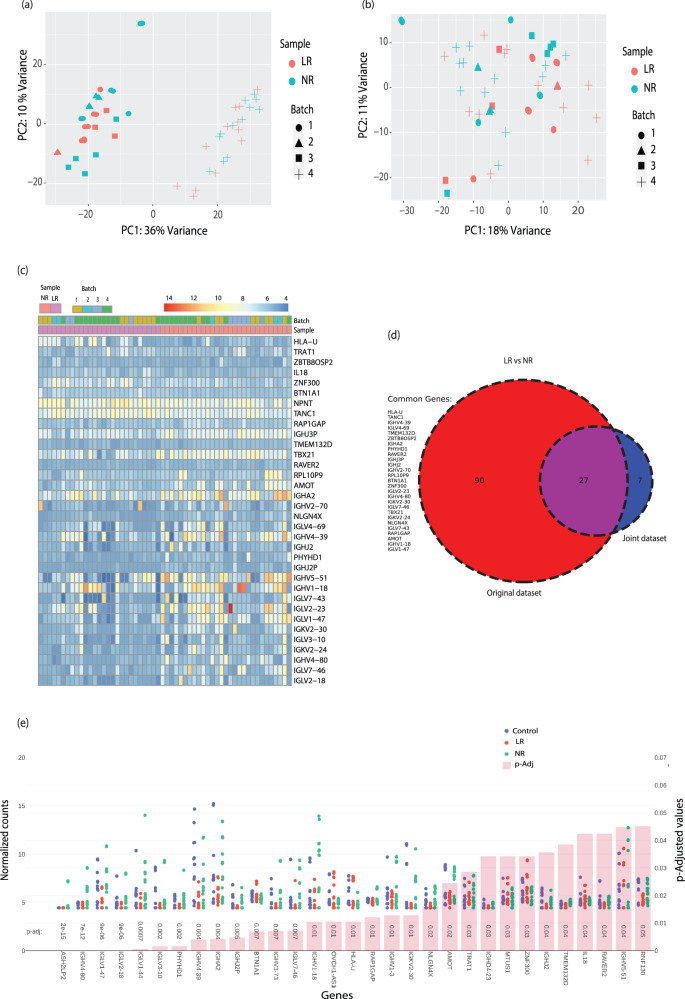
Differentially expressed genes between LR and NR LCLs are highly enriched in Immunoglobulin genes when adding a dataset from another study. **a** A PCA analysis of LR vs. NR LCLs’ RNA in the joint dataset (with an additional dataset from another lab, batch 4) before batch correction. Please note different colors are assigned for LR vs. NR samples & different shapes are assigned for different batches 1–4. LR RNA samples: 27; NR RNA samples: 29. 24 samples (5 LR in duplicates, 4 NR in duplicates, 3 control in duplicates) in Batch 1; 10 samples (1 LR in duplicate, 2 NR in duplicate, 2 control in duplicate) in Batch 2; and 12 samples in Batch 3 (3 LR-no replicate, 1 NR in duplicate plus 4 NR, 4 control-no replicate); Batch 4–24 samples (12 LR-no replicates and 12 NR-no replicates). In this figure, only LR and NR are plotted. **b** Same as (**a**) but after batch correction. **c** A heat map of the DEGs that reached a significance of FDR < 0.05 and fold-change >2 between BD LR and NR subtypes in their respective samples in the joint dataset. (LR RNA samples: 27; NR RNA samples: 29. RNA samples were sequenced in replicates for some of the subjects as mentioned above). **d** A Venn diagram showing 27 common DEGs between the original dataset and joint dataset of BD LR compared to BD NR samples, with a threshold of FDR < 0.05 and fold-change >2. **e** The plot counts of all significant DEGs in BD LR and BD NR samples compared to control samples arranged according to the ascending order of FDR (*p*-Adjusted) values < 0.05 with a fold-change >2 (joint dataset). The upper part of the graph shows the gene expression counts while the lower part of the graph presents the FDR values. (LR RNA samples: 27; NR RNA samples: 29. RNA samples were sequenced in replicates for some of the subjects. The expression counts have been normalized in DEseq2 in the Log2 scale).

To improve the statistical power, we next combined our in-house (original) data with the RNA-seq data from another cohort of BD LR and NR subtypes that was previously published [38] (also see Methods). As explained above, we repeated the analysis for batch effect. Since the data was acquired from a different laboratory, a higher magnitude of the batch effect was observed as visualized with the separate clustering of the first cohort (batches 1–3) in the left side of the PCA plot (quadrant 3) and the datasets from the second cohort (batch 4) at the right side of the PCA plot (quadrant 4) (Fig. 3a). This observed variability was reduced using our batch correction method. The PCA plots before and after batch corrections are presented in Fig. 3a & 3b respectively to show the effectiveness of the method we employed. A heat map showing the dysregulated genes with a fold change of over 2 between the LR and NR LCLs across different samples in different batches (batches 1–4) with an FDR < 0.05 is plotted in Fig. 3c. A Venn diagram showing the total of the shared 27 DEGs between the two datasets (original and joint) is shown in Fig. 3d. Figure 3e represents the plot counts of 31 DEGs for the joint dataset arranged according to the ascending p-Adj (FDR) values. We again found 80% of the top 10 DEGs belonging to the immunoglobulin family in this combined dataset coming from different laboratories and diagnosed by different psychiatrists (Fig. 3e). A volcano plot is presented in Supplementary Fig 3 for highly significant genes with a fold change of above 2 and FDR < 0.05 between LR vs. NR BD subtypes in the joint datasets.

### Shared genome-wide association studies (GWAS) genes

We were interested to see whether any of the DEGs in the LCLs in our original dataset also appeared in published GWAS of BD patients. We searched the GWAS catalog [59] for "bipolar/lithium" and extracted studies that reported significant genetic variants. We found overall 61 different studies but only 5 studies had one or more genetic variants in common with our DEGs from the original (batches 1–3) datasets after analysis (see the GitHub link for the code used). These genes along with the respective studies have been summarized in Table 1. Among the common genes, RIMS1 and BCL11B were notable because they appeared in 4 GWAS publications and appeared together in 2 of the publications. RIMS1 is a protein involved in neurotransmission because it is required for synaptic vesicle exocytosis [60]. RIMS1 has previously been found to have altered gene expression in cortical brain samples from SCZ and autism patients [61]. BCL11B (also known as CTIP2) is involved in both neuronal and immunological functions. BCL11B has been implicated in Alzheimer’s disease, Huntington’s disease, Neuro-HIV, learning, and memory, and its cellular role in cortical GABAergic neurons, medium spiny neurons, and vomeronasal sensory neurons have been studied [62]. In its non-neuronal role, BCL11B is crucial for T-cell differentiation and VDJ recombination in immunoglobulin proteins [62, 63]. ADCY1 and NPTX1 were also found to be associated with BD in our analysis as well as other GWAS studies [64, 65]. ADCY1 plays a potential role in learning and memory [66, 67], whereas NPTX1 is required for neural cell specification and is also known to be involved in synaptic plasticity [68, 69]. The other non-overlapping genetic variants from 56 genome-wide studies on BD subjects have been listed in Supplementary Table 1.

**Table 1:** Overlapping genes between GWAS data and DEGs in BD LR vs. NR LCLs.

Study Accession	Title	Overlapping genes	Total reported genes
GCST005081	Association of Polygenic Score for Schizophrenia and HLA Antigen and Inflammation Genes With Response to Lithium in Bipolar Affective Disorder: A Genome-Wide Association Study [101]	ADCY1	8
GCST008103	Genome-wide association study identifies 30 loci associated with bipolar disorder [102]	RIMS1, BCL11B, NPTX1	146
GCST009600	Genomic Relationships, Novel Loci, and Pleiotropic Mechanisms across Eight Psychiatric Disorders [103]	RIMS1, BCL11B	111
GCST011102	Novel Risk Loci Associated With Genetic Risk for Bipolar Disorder Among Han Chinese Individuals: A Genome-Wide Association Study and Meta-analysis [104]	RIMS1	23
GCST012465	Genome-wide association study of more than 40,000 bipolar disorder cases provides new insights into the underlying biology [105]	BCL11B	63
56 more studies that did not have any overlap with our set of DEGs are mentioned in Supplementary Table 1		Genes with no overlap with DEGS HOMER2, MIR52, NF1A, PTGFR, FAT, MHC, RP11–252P19.1,ADM,NFIA, DGKH NCAN, DPY19L3, CGNL1, SPATS2L, RND1, DDX23, CANCNB3, GRIK5, ST8SIA2, C15orf32, SLITRK1, SLITRK6, SNAP91, PRSS35, SDCCAG8, ANK3, CADM3, ENSG00000258081, POU3F2, MIR2113 FER1L5, LMAN2L, CNNM4, NF1A, ST6GALNAC3, ADCY2, ODZ4, TRANK1, ROR2, NFIL3, AUH, MIR3910, APOB, RPRD2, AC096669.1, LOC440300, LOC388152, LOC642423, GOLGA6L4, DNM1P41, GOLGA6L5, UBE2Q2P1,LOC100506874, ZSCAN2, SCAND2P, WDR73, NMB, SEC11A, ZNF592, ALPK3, SLC28A1OR4F16, OR4F29, LOC729467, LOC100133331, LOC100132287, FGGY, TMEM108, FAM178B, MKLN1, ETV5, FGGY, STAG1	

(Reference database: GWAS catalog [59]; see Methods) and the significant DEGs from the original dataset of BD LR and BD NR samples. The other studies that did not overlap with our set of DEGs are mentioned in Supplementary Fig. 1.

### Differentially expressed genes between BD and control LCLs are enriched in Immunoglobulin genes

**Fig. 4:**
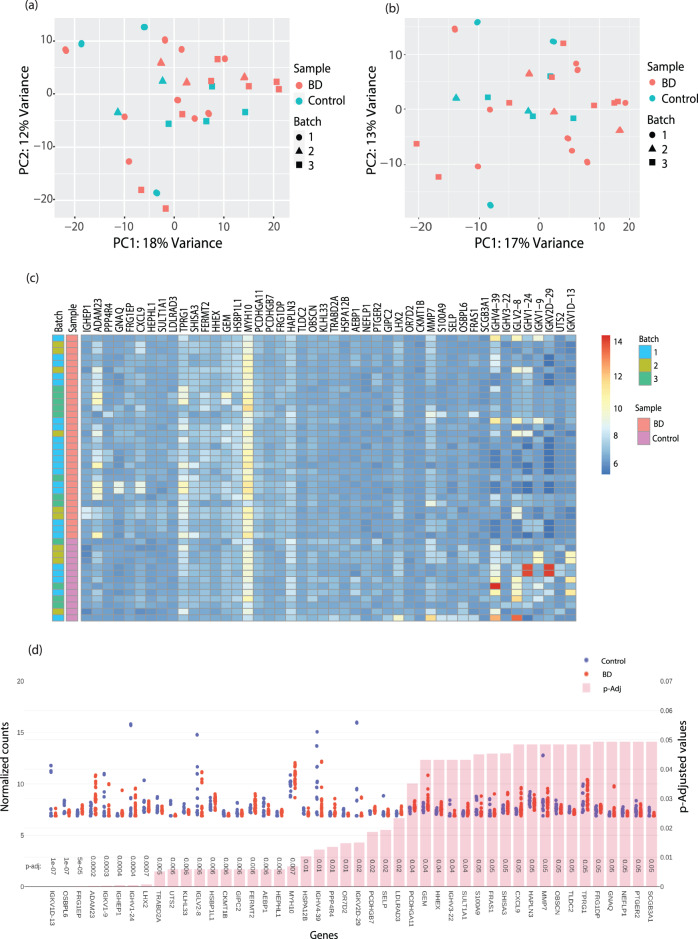
Differentially expressed genes between BD and control LCLs are enriched in Immunoglobulin genes. **a** PCA analysis of BD vs. control LCLs’ RNA in the original dataset before batch correction. (Please note different colors are assigned for BD vs. Ctrl samples & shapes for different batches 1–3. RNA samples: 32; Control RNA samples: 14. RNA samples were sequenced in replicates for some of the subjects as mentioned in Fig. 2a). **b** Same as (**a**) but after batch correction. **c** A heat map of the DEGs that reached a significance of FDR < 0.05 and fold-change >2 between BD and control groups in their respective samples in the original dataset (BD RNA samples: 32; Control RNA samples: 14. RNA samples were sequenced in replicates for some of the subjects.). **d** The plot counts of all significant DEGs in BD samples compared to control samples arranged according to the ascending order of FDR (*p*-Adjusted) values < 0.05 with a fold-change >2. (Original dataset). The upper part of the graph shows the gene expression counts while the lower part of the graph presents the FDR values. (RNA samples: 32; Control RNA samples: 14. The expression counts have been normalized in DEseq2 in the Log2 scale).

We next proceeded to identify genes that are differentially expressed between LCLs of BD patients compared to control individuals in the original dataset. We performed a similar analysis and compared the samples between the two groups- Control and BD (including LR and NR subtypes). The PCA plots before batch correction are presented in Fig. 4a. We again reduced the ambiguous technical factors by employing batch correction methods which is plotted in Fig. 4b. A heat map of the top DEGs with a fold change ≥2 and FDR < 0.05 is plotted from the original dataset (Batch 1, 2 & 3) (Fig. 4c). In Fig. 4d, we have presented the plot counts of the DEGs in ascending order of *p*-Adjusted values. A volcano plot is shown in Supplementary Fig 4a for the highly significant DEGs in BD compared to control groups.

We also analyzed to determine the fraction of immunoglobulin genes in the DEG groups vs. the total and substantially expressed immunoglobulin genes detected by RNA sequencing. First, we checked the percentage of immunoglobulin genes when observing just the highly expressed genes with two different thresholds of over a count of 100 (in the sequencing) or a count of 1000 (in the sequencing). As can be observed in Supplementary Fig. 4b (i–iii), the percentage of highly expressed immunoglobulin genes is ~1.1–1.2% in the LCLs. However, we found the immunoglobulin genes to be highly enriched in the BD vs control group (17.4%). The percentage of these genes was even higher in DEGs of LR vs. NR subtype comparisons in the original (29.1%) and in the joint datasets (52%). The graphs representing this data are presented in Supplementary Fig 4b (iv–vi). This evidence suggests the deregulation of the peripheral immune system in BD patients including the LR and NR subtypes compared to control.

### Predicting BD from the RNA of LCLs

**Fig. 5:**
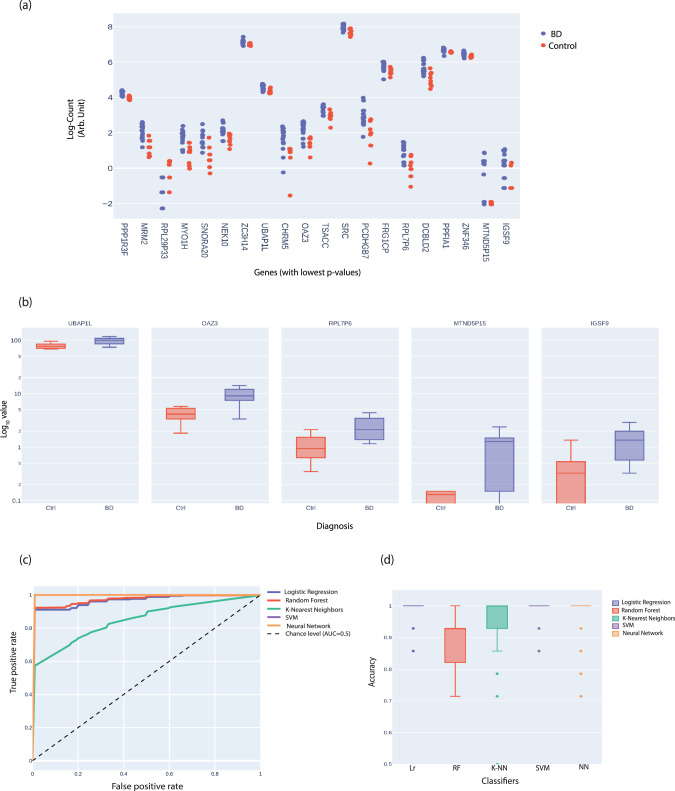
Predicting BD from the RNA of LCLs. Using 50%-50% cross-validation schemes, we utilized five different supervised classification algorithms to predict which of the LCLs’ RNA was extracted from a BD patient or control individual. Approximately 50% of the data (15 subjects) were used as a training set and ~50% (14 subjects) of the dataset were used as a test (out of the dataset that consisted of 19 BD and 10 controls) from cohort 1. The 50%-50% train/test approach was iterated 50 times. The five algorithms used were Logistic Regression (Lr), Random Forest (RF), Support Vector Machine (SVM), K-Nearest Neighbors (K-NN), and Neural Network (NN). **a** The gene expression counts of 20 genes in BD and control subjects with the lowest p-values (Mann Whitney U test) were used for the feature selection for the different classifiers. **b** Box plots of the expression of the five genes in BD and control groups that were used for the prediction. **c** A joint ROC for the prediction of BD vs. Control for the five classifiers. **d** The accuracy scores for BD vs. Control prediction for the five classifiers after splitting the data into test/train and repeating 50 times.

**Fig. 6:**
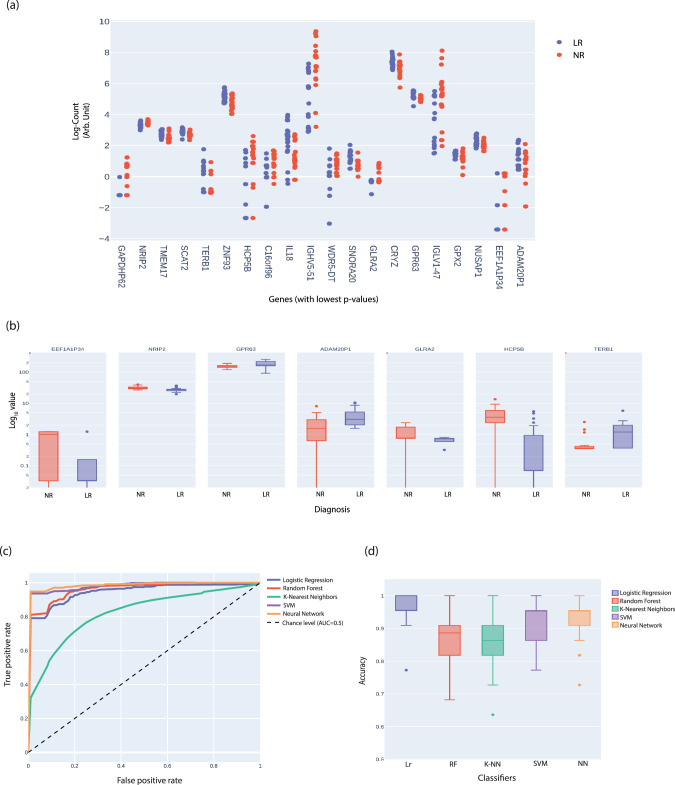
Predicting BD patients’ response to lithium treatment. Similarly as Fig. 5; Approximately 50% of the data (21 subjects) were used as a training set and ~50% (22 subjects) of the datasets were used as a test (out of the datasets that consisted of 19 BD subjects including LR & NR from cohort 1 & 24 BD subjects including LR & NR from cohort 2). **a** The plot counts of 20 genes in LR and NR subjects with the lowest *p*-values (Mann–Whitney *U* test) were used for the feature selection for the five classifiers. **b** Box plots of the expression of the seven genes in BD LR and NR subtypes that were used for the prediction. **c** A joint ROC for the prediction of LR vs. NR for the five classifiers. **d** Similarly, the accuracy scores of the classifiers for BD LR vs. NR prediction using a 50–50% cross-validation scheme repeated 50 times. Additionally, training was done on our dataset and the trained model was used to predict the lithium response of the BD patients in the Cohort 2 dataset (Milanesi et al. [38]).

We started this study to develop biomarkers that will be easy and fast to implement in the clinic. Batch effects are known to often drive the main differences between datasets of similar groups acquired in different technical conditions. It was therefore important for us to develop a robust protocol that will allow the prediction of BD (Fig. 5) and lithium response (Fig. 6) from the RNA-seq datasets. To examine this, we used five types of supervised classification algorithms: Logistic Regression (Lr), Random Forest (RF), K- Nearest Neighbors (K-NN), Support Vector Machine (SVM), and Neural Network (NN). As described in the Methods, to prevent overfitting we first performed batch correction on RNA-seq datasets using *Pycombat* and implemented a feature selection (see Methods for detail). We partitioned our data for cross-validation (see Methods). The expression values and the p-values of the subset of the 20 genes used for feature selection have been shown in Fig. 5a & Supplementary Fig 5a respectively. We selected a subset of the genes and ran the prediction algorithm (Lr) to see the dependence of the accuracy on the number of features (Supplementary Fig 5b). Notably, 5 features were enough to give an accuracy of about 93%. According to the algorithm, we selected a subset of 5 genes presented in (Fig. 5b) that were highly predictive for the disorder. Supplementary Fig 5c presents the confusion matrix (see Methods) of the 5 classifiers after repeating the split train /test approach 50 times. Figure 5c presents the performance of the respective 5 classifiers in the form of the area under the ROC curve. The Lr, SVM, and NN classifiers gave a successful prediction with a very low prediction error (AUC = 0.99 ± 0.00) (Fig. 5c). Additionally, other classifiers also gave a small error in the prediction: RF (AUC = 0.97 ± 0.04) and K-NN (AUC = 0.99 ± 0.01) (Fig.5c). Following randomly splitting the data into a train and test dataset and performing the classification 50 times, the accuracy of the prediction was also plotted in Fig. 5d. The Lr (0.996 ± 0.021), SVM (0.996 ± 0.022), NN (0.973 ± 0.058), and K-NN (0.956 ± 0.106) classifiers were more accurate than RF (0.888 ± 0.087%).

### Predicting BD patients’ response to lithium treatment

We next wondered whether the gene expression analysis from the LCLs obtained from the BD (LR & NR subtypes) and control subjects would be sufficient to predict lithium response and classify them into respective groups and subtypes. As described above, to reduce the noise and variation, we performed the batch correction and selected 7 genes to be utilized as features from the list of 20 genes with the lowest p-values (Fig. 6a & b; Supplementary Fig 6a & b) in a similar manner to what was described for selecting 5 genes to predict the disorder. Further, to test the robustness of our algorithms, we trained the above-mentioned five classifiers on the selected subset of 7 genes (Fig. 6b) using a 50-50 split train/test approach repeated 50 times (also see Methods). Supplementary Fig 6c presents the confusion matrix for 5 different classifiers. Figure 6c presents the ROC for the 5 classifiers (50% train, 50%test repeated 50 times for the datasets from batches 1–4). Among the predictors, Lr (AUC = 0.99 ± 0.01), SVM (AUC = 0.98 ± 0.03) & NN (AUC = 0.98 ± 0.03) showed a highly effective prediction with a lower error rate, but also RF (AUC = 0.96 ± 0.03) and K-NN (AUC = 0.95 ± 0.03) gave good results. Similarly, the accuracy of the classifiers has been plotted (Fig. 6d). The Lr (0.965 ± 0.038), NN 0.940 ± 0.052, and SVM (0.925 ± 0.063) had better accuracy compared to RF (0.874 ± 0.071%) and K-NN (0.86 ± 0.074) classifiers.

Additionally, for lithium response prediction, we trained the model on our dataset (9 LR and 10 NR) and used it to classify a completely different dataset that was acquired in another laboratory (batch 4) and on different patients [38]. This prediction was 95.8% accurate with SVM & NN and 91.7% accurate with the other three classifiers (RF, Lr, and K-NN).

## Discussion

The current understanding of the pathophysiology and progression of BD is inadequate. For better management of BD, biomarkers are necessary for diagnosis as well as for the selection of suitable therapeutic interventions [19, 24, 38].  Differential response to lithium treatment by more than half of BD patients is a conundrum in the field of BD research [8].  Some studies have provided information about a genetic link to non-response to lithium treatment, as family members of NR BD patients usually also do not respond to lithium [8, 70–72]. Nevertheless, mood episodes in BD are also known to be impacted by environmental factors like stress and traumatic experience [8].

In our study, we compared LCLs from 3 groups, Control, BD LRs, and BD NRs, by performing RNA sequencing to search for DEGs and associated biological pathways between the groups. The advantage of our method is its high-throughput, hypothesis-free approach, and the use of accessible patient-specific LCLs. LCLs have a huge potential for use in the discovery of biomarkers, especially in psychiatric disorders where the brain tissues are not accessible for molecular investigations from live patients [29, 38]. We have also analyzed and compared our in-house (original) dataset of BD LR and NR patient subtypes with a previously published study of 24 BD patients [38] (12 LR and 12 NR) as a joint dataset and found shared DEGs between the two cohorts. Additionally, in our study, we discovered significant DEGs between the BD and control groups.

When comparing the subtypes within the BD group, i.e., the LR vs. the NR patients, a set of genes belonging to the immunoglobulin heavy and light chains variable region were found to be significantly upregulated in lithium NRs as compared to LRs in the joint datasets. Increased expression of the immunoglobulin family genes specifically in lithium NRs entails the deregulation of the peripheral immune system. Apart from the immunoglobulin genes, we found the HLA-U, ZNF300, and TRAT1 genes to be significantly downregulated in NR subtypes. Interestingly, previous GWAS studies have reported the association of these genes with BD and other psychiatric disorders [73–75]. HLA-U is a pseudogene found on the MHC complex [76]. GWAS have revealed the MHC complex as an important risk gene in both BD and Schizophrenia (SCZ) [77–81]. Apart from its vital immunological role, the HLA locus has been proposed to play an important role during neurodevelopment [80, 81]. In our study, we found IL-18 to be significantly downregulated in NRs. IL-18 is a proinflammatory cytokine reported to be expressed in different regions of the brain and has a known role in neuroinflammation [82]. Some studies have reported high levels of circulating IL-18 in BD and SCZ patients [83–85]. The altered expression of these genes specifically in NRs compared to LRs is intriguing, especially because these genes overlap with previous reports in BD.

Further, when we analyzed the original and joint datasets, 27 genes were found to be common. Apart from HLA-U and IGV genes, TANC1 and TMEM 132D were among the top 5 most common genes. The TANC1 protein has been shown to interact with PSD95 as well as other synaptic proteins such as glutamate receptors [86]. A TANC1 mutation, along with mutations in NRXN1 and RBMS1 genes, were implicated in psychomotor delayed development in a case study of chromosomal inversion [87]. GWAS have linked the TMEM 132D polymorphism to anxiety and panic disorder [88, 89]. TMEM 132D has also been reported to be dysregulated at the mRNA level in brain regions associated with anxiety disorders [89]. The differential expression of these critical genes is even more intriguing since we integrated datasets from another group that was conducted in a different cohort.

We also compared gene expression between BD and control LCLs. We found a downregulation of immunoglobulin kappa chain variable genes in BD patients when compared with control groups. ADAM23, IGHEP1, GNAQ, FRG1EP, and PPP4R4 were upregulated in BD patients, including LRs and NRs as subsets. GNAQ and ADAM23 have earlier been reported to be associated with BD [90, 91]. ADAM23 was previously reported in a microarray-based gene expression study of BD postmortem brain samples [91]. ADAM23 is a membrane protein belonging to the family of ADAM proteins and is part of a presynaptic complex interacting with the Lgl receptor [92]. While FRG1EP has a reported function in cancer, the function of the other above-mentioned genes is not well understood, especially in the context of psychiatric diseases [93].

Overall, our findings suggest significant dysregulation of genes with immunological function as well as alteration of genes involved in synaptic pathways in BD LCLs. Overwhelming reports have highlighted the importance of immune dysregulation in BD and SCZ [94–97]. We also found genes implicated in neuro-inflammation and anxiety disorder altered in BD LCLs. Furthermore, between BD patient subtypes, we found cytokines and immunological genes specifically altered in NR compared to LR patients.

We used five diverse supervised classification algorithms – Lr, RF, K-NN, SVM, and NN to predict BD and lithium treatment response. Each classifier has its own set of pros and cons as described briefly above (in Methods) and reviewed previously [36, 55, 57, 98]. One typical drawback with supervised classification algorithms is that their performance decreases with high-dimensional datasets (like RNA-seq data) with a large number of features leading to overfitting [98, 99]. To prevent overfitting, we reduced the number of genes (features) used for training the classifier to only 5 & 7 (for BD prediction and lithium response respectively) and thus reduced the complexity of the model by selecting only the statistically informative and robust genes for prediction. Further, our model used a random sampling cross-validation scheme to split the data into training and test datasets. This gave robust statistical results with a very low error rate especially using the Lr, SVM, and NN classifiers for lithium response prediction. The error rate was very low (less than 5% with SVM and NN; approx. 8% for Lr, RF, and K-NN) even when trained on our in-house dataset and tested on the data from another laboratory. The BD samples could also be predicted with high conformity and distinguished from the control samples with minimal errors using the Lr, SVM, and NN classifiers.

Some studies have used supervised machine learning algorithms for Diabetes, Cardiovascular diseases, Cancers, Alzheimer’s disease, and Parkinson’s disease using clinical parameters and neuroimaging datasets [100]. Ours is the first study to use transcriptomic datasets from LCLs of BD patients to predict the disorder as well as the responsiveness to lithium. The method is cost-effective and scalable and can be easily implemented in a psychiatric clinic. While one of the limitations of the study is the insufficient clinical sample size given the complexity and polygenic nature of BD, we showed the robustness of our algorithm by testing it on a new dataset from another lab. Further, the use of transformed B cell lines (LCLs) may not be the ideal tissue choice for a neuropsychiatric disorder like BD but the fact that we could find statistically significant DEGs and were able to predict and classify BD subtypes using the classifiers provides proof about the existing specific genetic signatures between BD subtypes.

In conclusion, using RNA-seq, we have found a set of DEGs from LCLs of BD patients that can be used as potential biomarkers to diagnose as well as classify BD patient subtypes. Functional studies of these genes in model systems should also aid in elucidating the cellular and molecular processes underlying BD pathophysiology. Importantly, we hope that our study and our developed algorithm will serve as an easy and ready-to-use protocol for deciding on effective treatment in the clinic within days of diagnosis.

### Supplementary information

Supplementary Figures 1-6

Supplementary Table 1

## Data Availability

The data including raw files are available in the following Github link below. All the codes used in this study for RNA-seq data analysis, Machine learning predictor analysis, and GWAS analysis are also available in the following link. https://github.com/Precision-Disease-Modeling-Lab/Lithum-Respose-Predictor.
