# Supplementary material for: Immunoglobulin genes expressed in lymphoblastoid cell lines discern and predict lithium response in bipolar disorder patients
Source: Mol Psychiatry. 2023 Jul 24;28(10):4280–93. doi: 10.1038/s41380-023-02183-z (PMC10827667; doi:10.1038/s41380-023-02183-z)

Supplementary Fig.1. Representative images for the culture of the LCLs from (a) control, (b) BD LR and (c) BD NR. As mentioned in th methods these cells were derived from PBMCs after transformation with EBV . The LCLs grow in suspension cultures as aggregates of transformed B cells (Scale bar: 20uM).

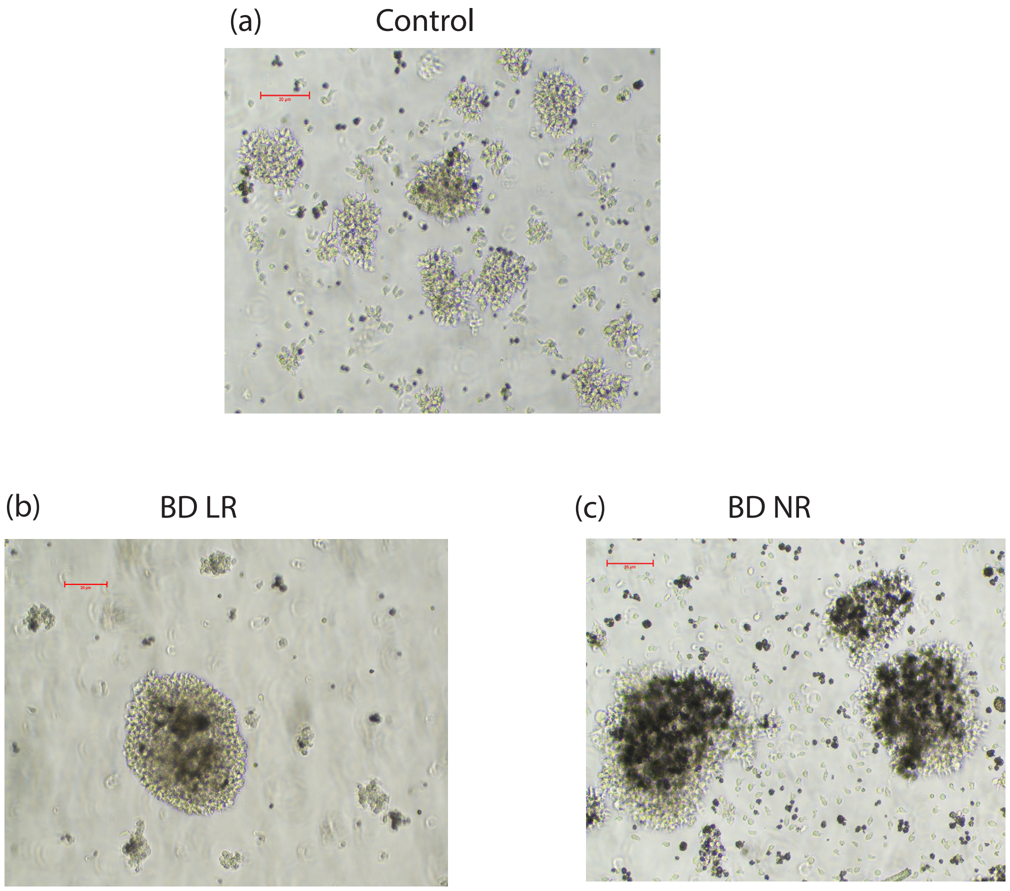

Supplementary Fig .2. Plot count of genes differentially expressed in BD LR vs BD NR LCL RNA in original datset (cohort 1) arranged in ascending order of p-Adj values (a) & (b) respectively (Continued from Fig.2d)

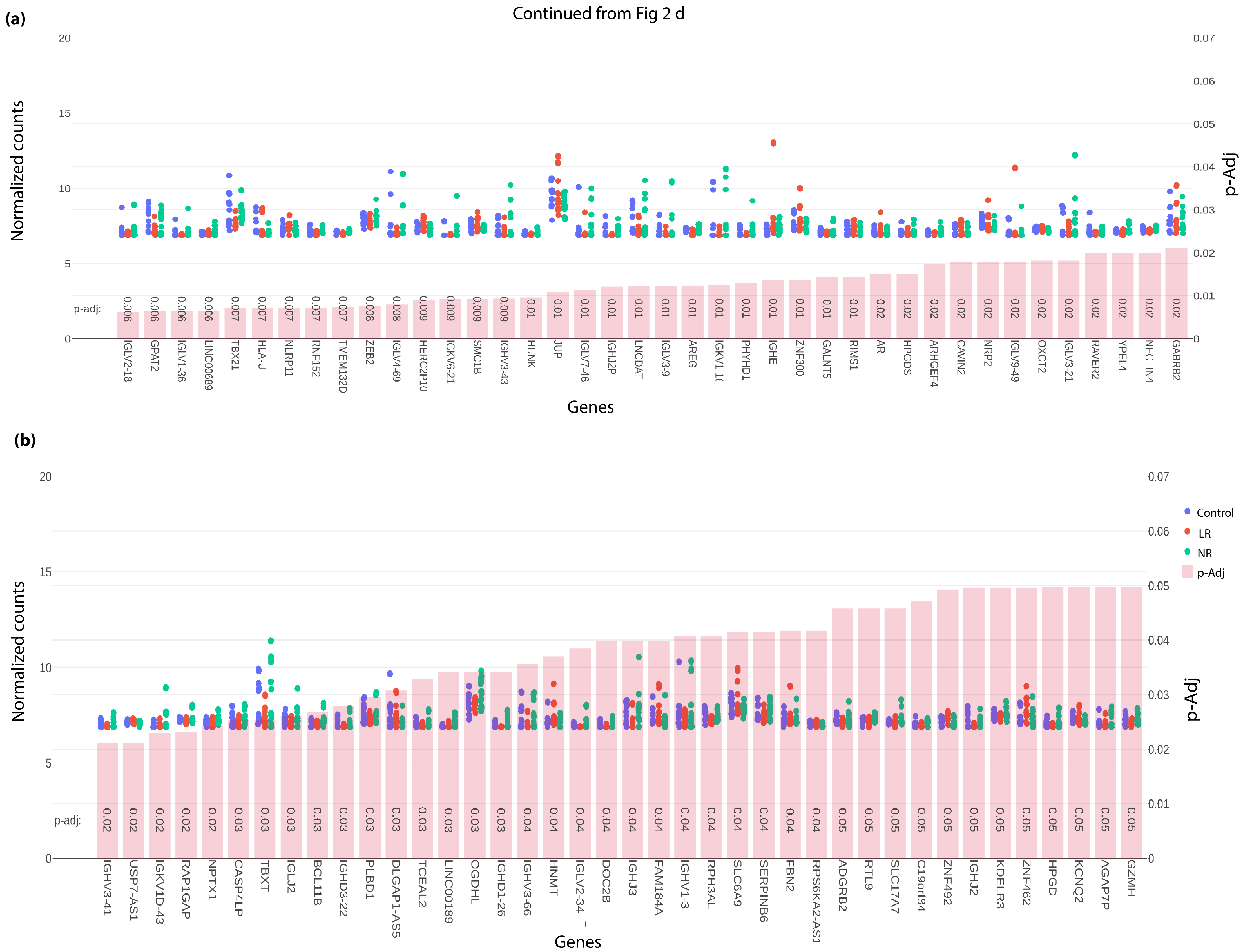

Supplementary Fig.2c A volcano plot of DEGs with a fold-change (FC) cutoff of 2.0 and a FDR cut-off of 0.05 for LR vs NR in original datset

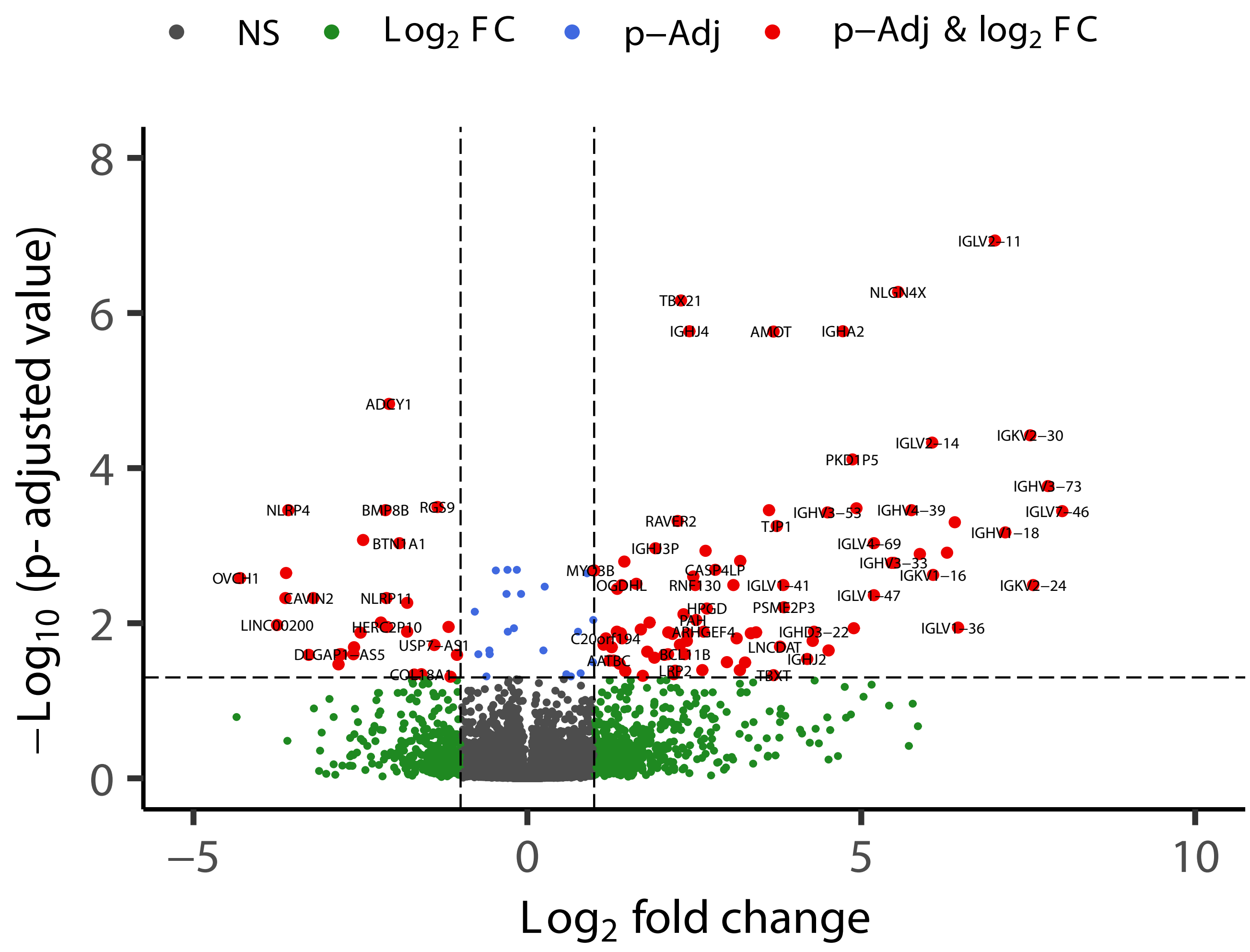

FC cutoff, 2.0; p–adj cutoff, 0.05

Grey: NS- Genes that were not significant DEGS;
Green:Log2 FC- Genes that had only fold change of 2;
Blue: genes with only p-Adj (FDR) value of 0.05 but no log2 FC calculated from DESeq2 (see methods);
Red: Genes that had both Log2 FC and pAdj<0.05 (FDR cut off)

Suppementary Fig.3 A volcano plot of DEGs with a fold-change (FC) cutoff of 2.0 and a FDR cut-off of 0.05 in LR vs NR in joint datset

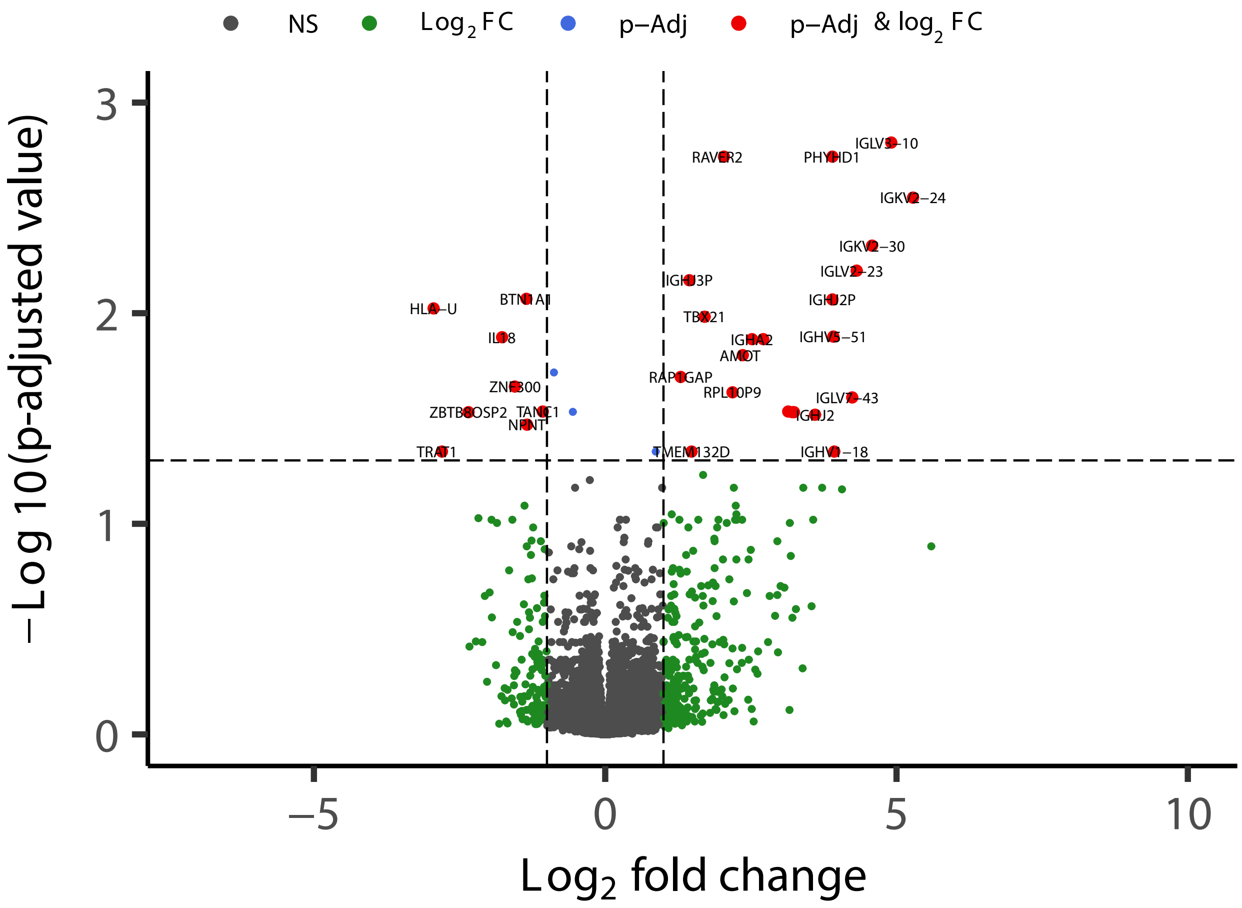

Grey: NS- Genes that were not significant DEGS;
Green:Log2 FC- Genes that had only fold change of 2;
Blue: genes with only p-Adj (FDR) value of 0.05 but no log2 FC calculated from DESeq2 (see methods);
Red: Genes that had both Log2 FC and pAdj<0.05 (FDR cut off)

Supplementary Fig 4

(a) A volcano plot of DEGs with a fold-change (FC) cutoff of 2.0 and a FDR cut-off of 0.05 in BD vs. control in the orginal datasets.

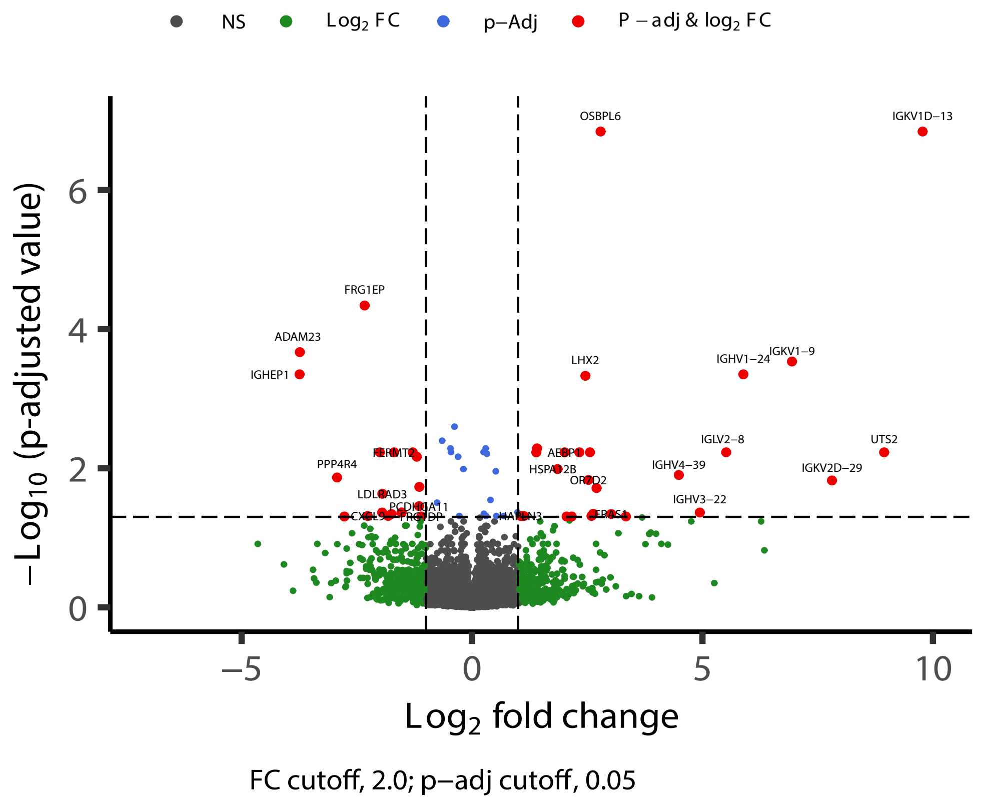

Grey: NS- Genes that were not significant DEGS;
Green: Log2 FC- Genes that had only fold change of 2;
Blue: genes with only p-Adj (FDR) value of 0.05 but no log2 FC calculated from DESeq2 (see methods);
Red: Genes that had both Log2 FC and pAdj<0.05 (FDR cut off)

(b) Percentage of IGG genes (yellow circle) are enriched in (iv) BD vs control DEGs as well as (v) & (vi) LR vs NR DEGs in comparison to (i-iii) Percentage of total IGG genes detected via RNA seq or genes with atleast 100 or 1000 counts

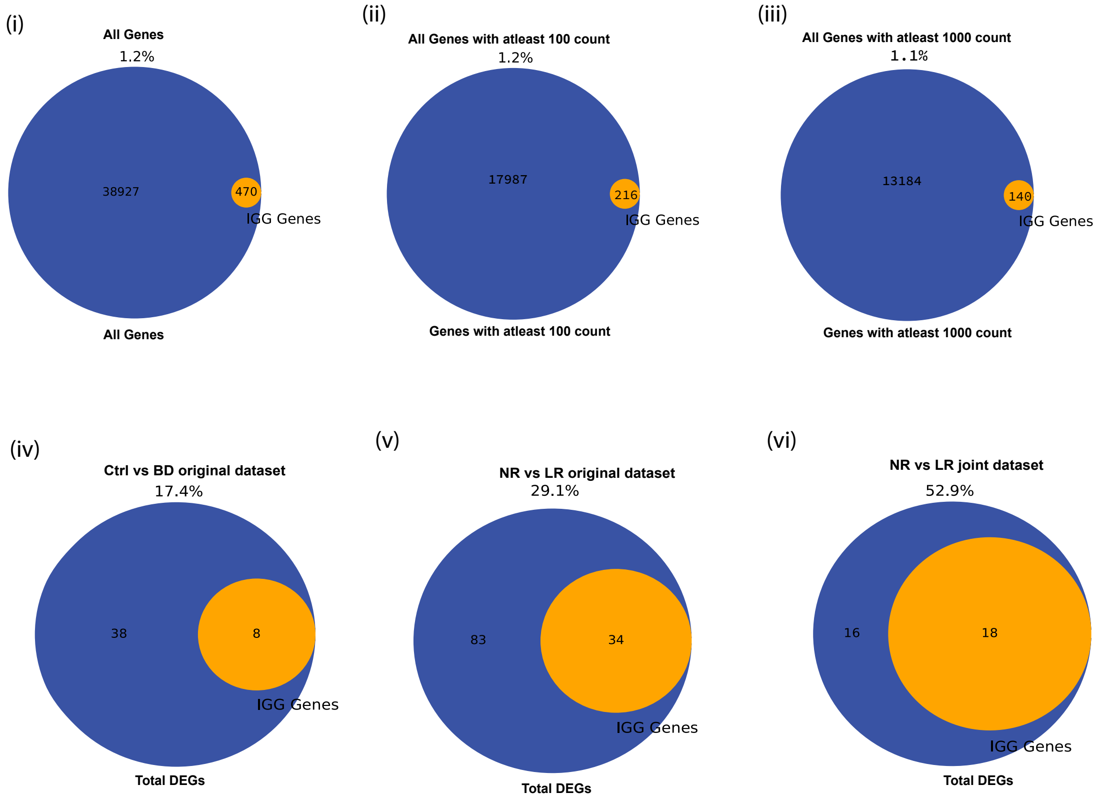

Supplementary Fig .5.

(a) Bar graph for the corresponding p-values of the 20 genes used for feature selection in BD vs Control predictor analysis (plotted in Fig 5a)

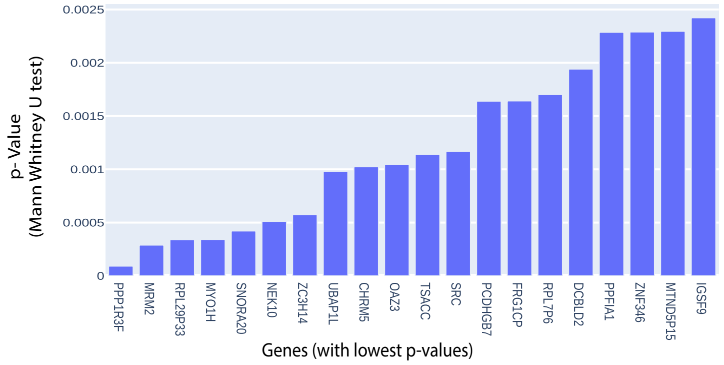

(b) Graph showing the logistic regression *accuracy* vs *number of features* for features selection with accuracy greater than 0.95 for BD vs Control

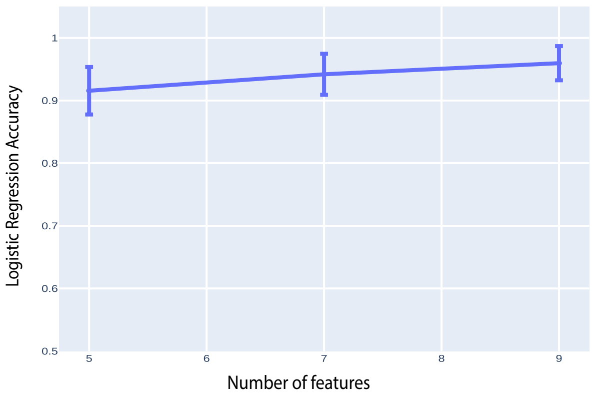

(c) Confusion matrix for all the 5 classifiers used for predicion of BD vs Control predictor analysis using above features for 50% (14 subjects) of the datasets which comprised of 19 subjects (LR & NR) and 10 controls from cohort 1. The 50%-50% train/test approach was repeated 50 times.

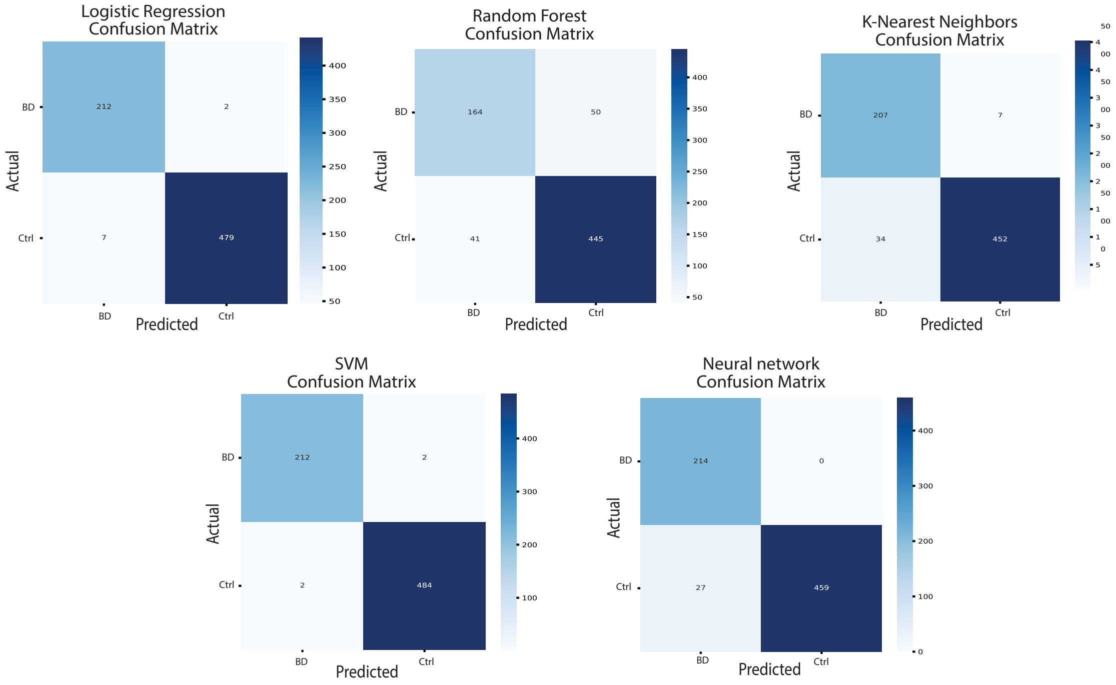

Supplementary Fig .6.

(a) Bar graph for the corresponding p-values of the 20 genes used for feature selection in BD subtypes LR vs NR predictor analysis (plotted in Fig 6)

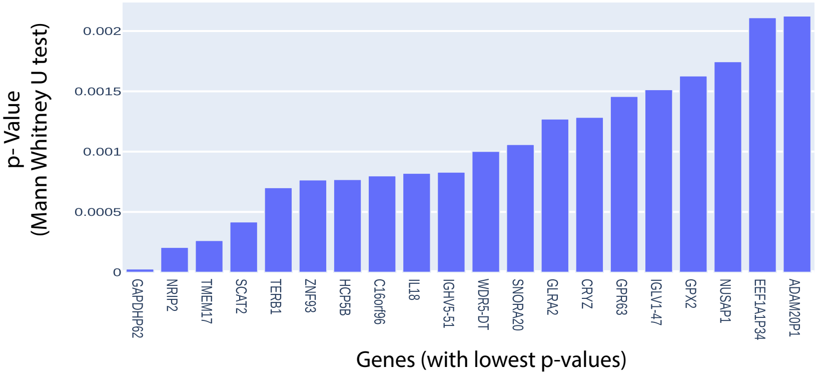

(b) Graph showing the *accuracy* vs *number of features* for features selection with accuracy greater than 0.95 for BD subtypes- LR vs NR

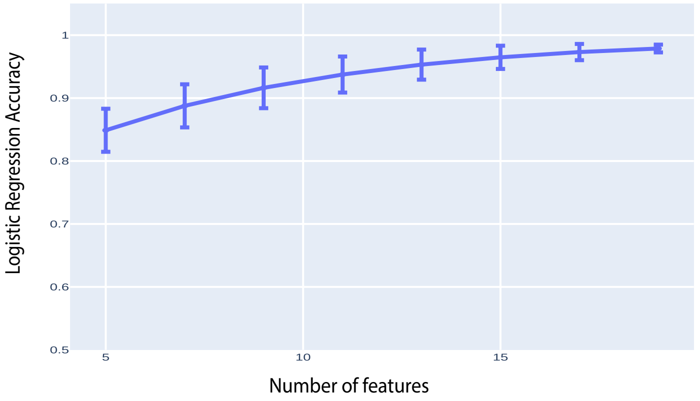

(c) Confusion matrix for all the 5 classifiers used for predicion of LR vs NR predictor analysis using above features for 50% (22 subjects) of the datasets which comprised of 19 subjects (LR & NR) from cohort1+ 24 (LR & NR) from cohort2=43 total subjects. The 50%-50% train/test approach was repeated 50 times.

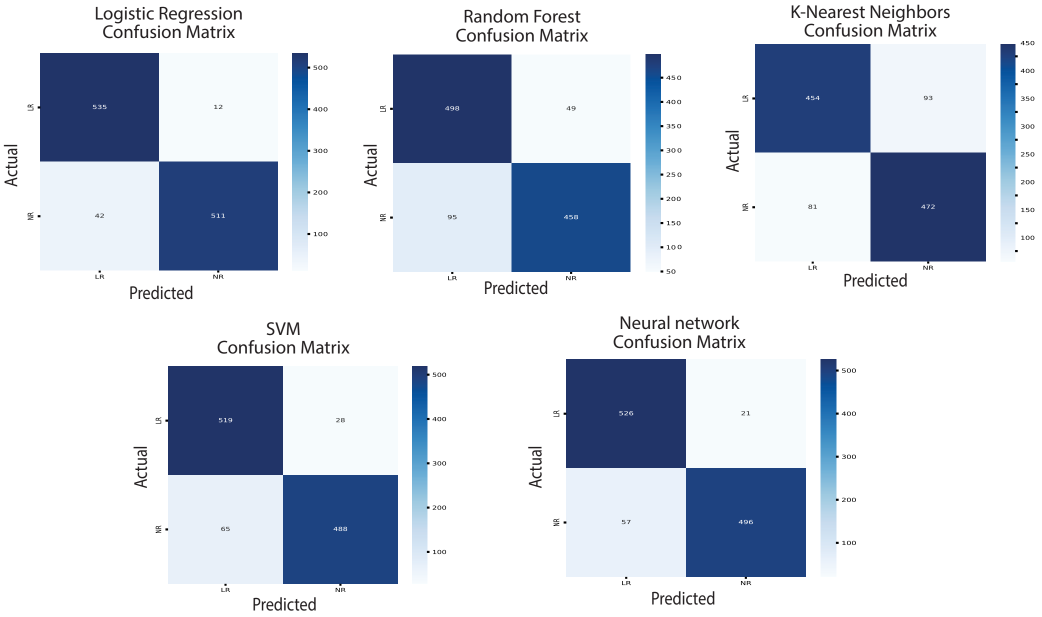

Supplement: Supplementary file 1 — Supplementary Figures 1-6 [file 41380_2023_2183_MOESM1_ESM.pdf]
